# Screening and identification of significant media components using Plackett–Burman design for enhanced extracellular protease production by *Bacillus* sp. MTCC 511 under mutagenic stress conditions

**DOI:** 10.1186/s40643-026-01134-0

**Published:** 2026-10-08

**Authors:** Amarish Kumar Sharma, Ajay Kumar, Manoj Kumar Jena

**Affiliations:** 1 Department of Biotechnology, Parul Institute of Technology, Parul University, Vadodara, Gujarat 390761 India; 2 Department of Bioengineering and Biosciences, Lovely Professional University, Phagwara, Punjab 144411 India

**Keywords:** Extracellular protease, *Bacillus* sp., Plackett–Burman design, Factor screening, Bioprocess engineering, Mutagenesis

## Abstract

**Background:**

Statistical screening of media components is essential for identifying the key variables that govern protease yield from *Bacillus* species. We used a 12-run Plackett–Burman Design (PBD) to screen ten independent variables—dipotassium phosphate, monopotassium phosphate, ammonium sulphate, sodium nitrate, magnesium sulphate heptahydrate, calcium chloride, carbon source, nitrogen source, agitation speed, and pH—for their effects on extracellular protease production by *Bacillus* sp. MTCC 511 under four mutagenic stress conditions: thermal treatment at 70 °C and 80 °C (10 min each) and UV irradiation (2 and 3 min).

**Results:**

Enzyme activity was quantified by Folin–Ciocalteu assay against a tyrosine standard (R2 = 0.998). The highest protease activity, 8.66 U/mL, was recorded under 3-min UV irradiation in a medium containing starch (1%), yeast extract (0.1%), K_2_HPO_4_ (0.05 M), KH_2_PO_4_ (0.05 M), (NH_4_)_2_SO_4_ (0.05 M), NaNO_3_ (0.1 M), MgSO_4_·7H_2_O (0.002 M), and CaCl_2_ (0.002 M) at pH 9 and 120 rpm, representing a 20.6-fold improvement over the unsupplemented basal medium. ANOVA showed R2 > 97% for the 70 °C, 80 °C, and UV-3 min models; the UV-2 min model was statistically inadequate (R2 = 56.52%). Pareto analysis pointed to agitation speed, NaNO_3_, MgSO_4_·7H_2_O, and carbon source as the most influential factors. Pearson correlation between thermal and UV-3 min responses (r = 0.72–0.83) is consistent with previously described σ^B^-dependent stress–response pathways.

**Conclusions:**

The PBD screening efficiently identified significant factors whose effects are consistent with established regulatory mechanisms including nitrogen catabolite repression, carbon catabolite repression, and dissolved oxygen-dependent secretion. Follow-up response surface optimization is warranted to characterize factor interactions and identify the global optimum.

**Graphical abstract:**

**Figure:**
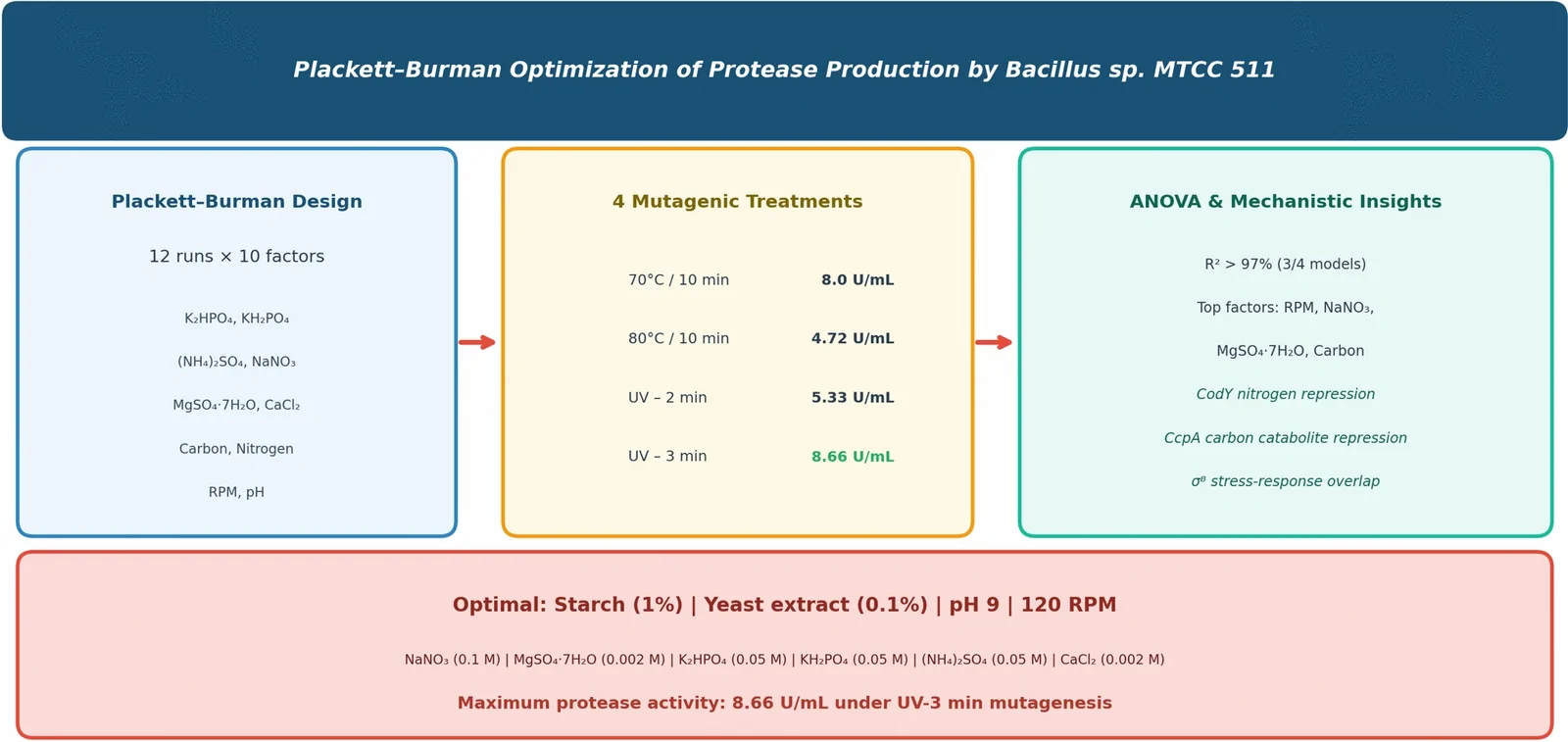

## Background

Proteases (EC 3.4) represent the single largest class of industrial enzymes, accounting for roughly 60% of the worldwide enzyme market (Rao et al. 1998; Anwar and Saleemuddin 1998; Gurumallesh et al. 2019). They hydrolyze peptide bonds in protein substrates and serve a wide range of industrial applications—from detergent formulation and food processing to leather treatment and pharmaceutical manufacturing (Rao et al. 1998; Kumar and Takagi 1999). Growing demand for proteases that are thermostable, alkali-tolerant, and compatible with surfactants continues to drive research into microbial production systems (Anwar and Saleemuddin 1998; Prakasham et al. 2006).

*Bacillus* species dominate commercial alkaline protease production worldwide (Kumar and Takagi 1999; Gupta et al. 2002). Their appeal lies in high extracellular enzyme titres, a well-understood Sec-dependent secretion system, genetic tractability, and GRAS status for several species (Kumar and Takagi 1999; Durham et al. 1987). Industrial proteases such as subtilisin from *B. subtilis* and *B. licheniformis* remain the backbone of modern detergent formulations (Rao et al. 1998).

At the physiological level, extracellular protease synthesis in *Bacillus* responds to a web of regulatory inputs. Carbon catabolite repression (CCR), operating through the CcpA-HPr (Ser-P) complex and catabolite-responsive elements (cre), dampens protease transcription when fast-metabolizing sugars like glucose are present (Deutscher 2008). Nitrogen catabolite repression (NCR), mediated primarily by the global regulator CodY, suppresses the *aprE* and *nprE* promoters when preferred nitrogen sources are plentiful (Kumar and Takagi 1999; Strauch and Hoch 1993). The DegS–DegU two-component system and the Spo0A phosphorelay further coordinate the shift toward extracellular enzyme production during the transition from exponential to stationary phase (Strauch and Hoch 1993; Msadek et al. 1998a).

Classical “one-variable-at-a-time” (OVAT) optimization ignores interactions among variables and demands a prohibitive number of experiments (Beg et al. 2003). Plackett–Burman Design (PBD), a two-level fractional factorial approach, screens many variables simultaneously in few runs, estimating main effects without confounding with two-factor interactions (Beg et al. 2003; Saxena and Singh 2010; Romsomsaa et al. 2010). PBD has been applied to protease optimization in *B. mojavensis* (Beg et al. 2003), *B. subtilis* (Romsomsaa et al. 2010; Navaneeth et al. 2009), and *B. cereus* (Shafee et al. 2005).

Mutagenic treatments—thermal shock and UV irradiation—are classical strain-improvement tools that can generate heritable changes affecting enzyme output. UV light induces cyclobutane pyrimidine dimers, triggering the SOS response and error-prone DNA repair (Das and Prasad 2010). Thermal stress activates the σ^B^-dependent general stress regulon, which governs over 150 genes in *Bacillus*, some of which participate in protein quality control and extracellular secretion (Haldenwang 1995; Völker et al. 1994). Divalent cations (Ca^2+^, Mg^2+^, Mn^2+^) can stabilize protease structure at elevated temperatures (Akel et al. 2009; Kaur et al. 2001), yet excess metal ions sometimes suppress secretion, making concentration optimization important (Uyar et al. 2011). Emon et al. (2024) recently demonstrated enhanced protease production from a UV-mutant B. subtilis strain using consolidated bioprocessing.

Despite the extensive literature on PBD-based protease screening in Bacillus, no study to our knowledge has compared the enzyme response across multiple mutagenic treatments (thermal and UV) within a single PBD framework, nor has Pearson correlation analysis been used to probe whether different physical stressors share overlapping regulatory pathways. The present work addresses this gap by: (1) conducting a systematic side-by-side comparison of four distinct mutagenic conditions within a unified 12-run PBD matrix; (2) applying inter-treatment correlation analysis to infer shared stress–response regulation; and (3) linking the observed factor effects to established physiological models in Bacillus as testable hypotheses for future molecular studies. The thermal treatment temperatures (70 °C and 80 °C) were selected on the basis of preliminary kill-curve experiments indicating sub-lethal stress within this range, while the UV exposure durations (2 and 3 min) were chosen from published mutagenesis protocols for Bacillus (Ahmed et al. 2010; Das and Prasad 2010).

## Methods

### Microorganism and culture maintenance

*Bacillus* sp. MTCC 511 was obtained from the Microbial Type Culture Collection and Gene Bank (MTCC), IMTECH, Chandigarh, India. The lyophilized culture was revived in nutrient broth (HiMedia, Mumbai) at 37 °C for 24 h. Stock cultures were kept in nutrient broth (pH 7.8) with 20% (v/v) glycerol at − 20 °C; working cultures were sub-cultured on nutrient agar slants stored at 4 °C.

### Chemicals

Ammonium sulphate, beef extract, calcium chloride, casein, dipotassium phosphate, glucose, magnesium sulphate heptahydrate, monopotassium phosphate, trichloroacetic acid (TCA), soluble starch, sodium nitrate, yeast extract, sodium carbonate, L-tyrosine, and Folin–Ciocalteu reagent were all analytical grade (HiMedia; Qualigens; Loba Chemie, India). Solutions were made in double-distilled water and autoclaved (121 °C, 15 min, 15 psi).

### Protease production

Fermentation was carried out in a 5 L stirred-tank bioreactor (3.5 L working volume). Basal nutrient broth (pH 7.8) was supplemented with: (NH_4_)_2_SO_4_ (0.01–0.05 M), glucose or starch (1% w/v), CaCl_2_ (0.002–0.006 M), K_2_HPO_4_ (0.01–0.05 M), beef extract (1% w/v) or yeast extract (0.1% w/v), MgSO_4_·7H_2_O (0.002–0.006 M), KH_2_PO_4_ (0.01–0.05 M), and NaNO_3_ (0.1–0.5 M). After sterilization the medium was inoculated with 5% (v/v) seed culture and incubated at 48 °C with agitation at 80–120 rpm for 24 h. The 48 °C temperature was selected from preliminary trials as conducive for thermotolerant protease expression by this strain. Culture broth was centrifuged at 1200×*g* for 15 min at 37 °C; this speed was verified in preliminary runs to yield a clear supernatant (OD₆₀₀ < 0.02) adequate for colorimetric analysis. The cell-free supernatant served as crude enzyme.

### Mutagenic treatments

Four treatments were applied to overnight cultures (OD_600_ ~ 0.8): (1) 70 °C for 10 min (water bath); (2) 80 °C for 10 min; (3) UV-254 nm for 2 min (germicidal lamp, 30 cm distance); (4) UV-254 nm for 3 min. Surviving cells were immediately inoculated into the 12 PBD media and incubated at 37 °C for 24 h.

Validation fermentations were conducted at 37 °C rather than 48 °C to accommodate the potentially reduced thermotolerance of mutagenized cells.

### Plackett–Burman design

A 12-run PBD screened ten variables at two coded levels (Table 1). The first-order model Y = β_o_ + ∑β_i_X_i_ was fitted, where Y is enzyme activity (U/mL), β_o_ the grand mean, β_i_ the coefficient for each factor, and X_i_ the coded level. The 12 experimental runs were conducted in randomized order as generated by MINITAB version 16 (Minitab Inc., State College, PA, USA). Main effects were calculated as the difference between average responses at the high and low levels of each variable.

**Table 1:** Independent variables and their coded levels in the Plackett–Burman design

Code	Variable	Low (− 1)	High (+ 1)	Low level	High level
C1	K_2_HPO_4_	− 1	+ 1	0.01 M	0.05 M
C2	KH_2_PO_4_	− 1	+ 1	0.01 M	0.05 M
C3	(NH_4_)_2_SO_4_	− 1	+ 1	0.01 M	0.05 M
C4	NaNO_3_	− 1	+ 1	0.1 M	0.5 M
C5	MgSO_4_·7H_2_O	− 1	+ 1	0.002 M	0.006 M
C6	CaCl_2_	− 1	+ 1	0.002 M	0.006 M
C7	Carbon source	Glucose	Starch	1%	1%
C8	Nitrogen source	Beef extract	Yeast extract	1%	0.1%
C9	Agitation speed	80 rpm	120 rpm	–	–
C10	pH	5	9	–	–

### Protease activity assay

A tyrosine calibration curve (0–65 µg/mL) was prepared at 660 nm with sodium carbonate and Folin–Ciocalteu reagent (37 °C, 30 min). Protease activity was measured by the modified Folin–Ciocalteu method (Huang et al. 2003): cell-free supernatant was mixed with 0.1% (w/v) casein in glycine–NaOH buffer (pH 10) and incubated at 37 °C for 10 min. The reaction was stopped with 110 mM TCA, left to stand for 10 min, filtered (Whatman No. 1), and the filtrate was treated with sodium carbonate and Folin–Ciocalteu reagent (37 °C, 30 min) before reading at 660 nm. One unit (U) = 1 µg tyrosine released mL⁻1 min⁻1. All measurements were performed as three technical replicates from the same culture supernatant.

### Experimental validation

All 12 trial runs of the PBD were independently validated in a 15 L stirred-tank bioreactor (11 L working volume). pH was held at 5 (runs 1, 4, 5, 7, 8, 11, 12) or 9 (runs 2, 3, 6, 9, 10). Medium was sterilized, inoculated with 5% seed culture, and fermented at 37 °C for 24 h at 120 rpm (runs 1, 2, 3, 7, 10, 12) or 80 rpm (runs 4, 5, 6, 8, 9, 11). Dissolved oxygen was maintained at 25% saturation. Samples were drawn every 3 h. The consensus optimal conditions were identified post hoc from the direction of main effects across the three statistically adequate models.

### Statistical analysis

ANOVA, main-effect estimation, and regression analysis were performed in MINITAB version 16. Factor significance was assessed by Student’s t-test; model adequacy by Fisher’s F-test, R2, and adjusted R2. Percentage contributions were computed as factor SS/total SS. Pareto analysis ranked factors by variance contribution. Pearson correlation coefficients (r) were calculated between treatment responses.

## Results and discussion

### Tyrosine standard curve

**Fig. 1:**
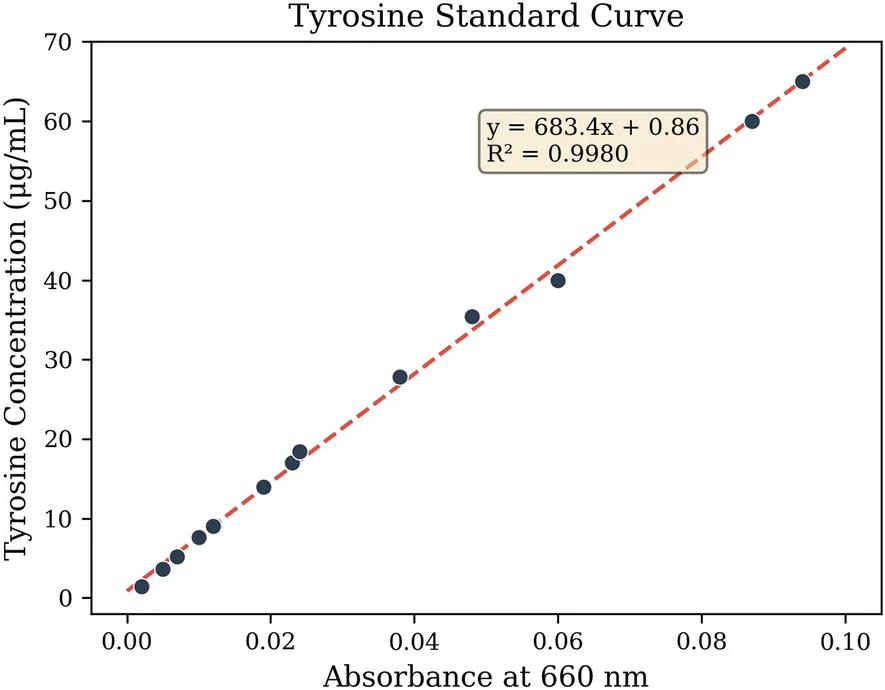
Tyrosine standard curve for protease activity determination. The linear regression equation y = 683.4x + 0.86 (R2 = 0.998) was used to convert absorbance values at 660 nm to tyrosine concentration (µg/mL). Error bars represent standard deviation of three technical replicates

The calibration curve was linear over 1.4–65.0 µg/mL, with a regression equation of y = 683.4x + 0.86 and R2 = 0.998 (Fig. 1), confirming the suitability of the assay for quantifying protease activity.

### Effect of mutagenic treatments on protease production

**Fig. 2:**
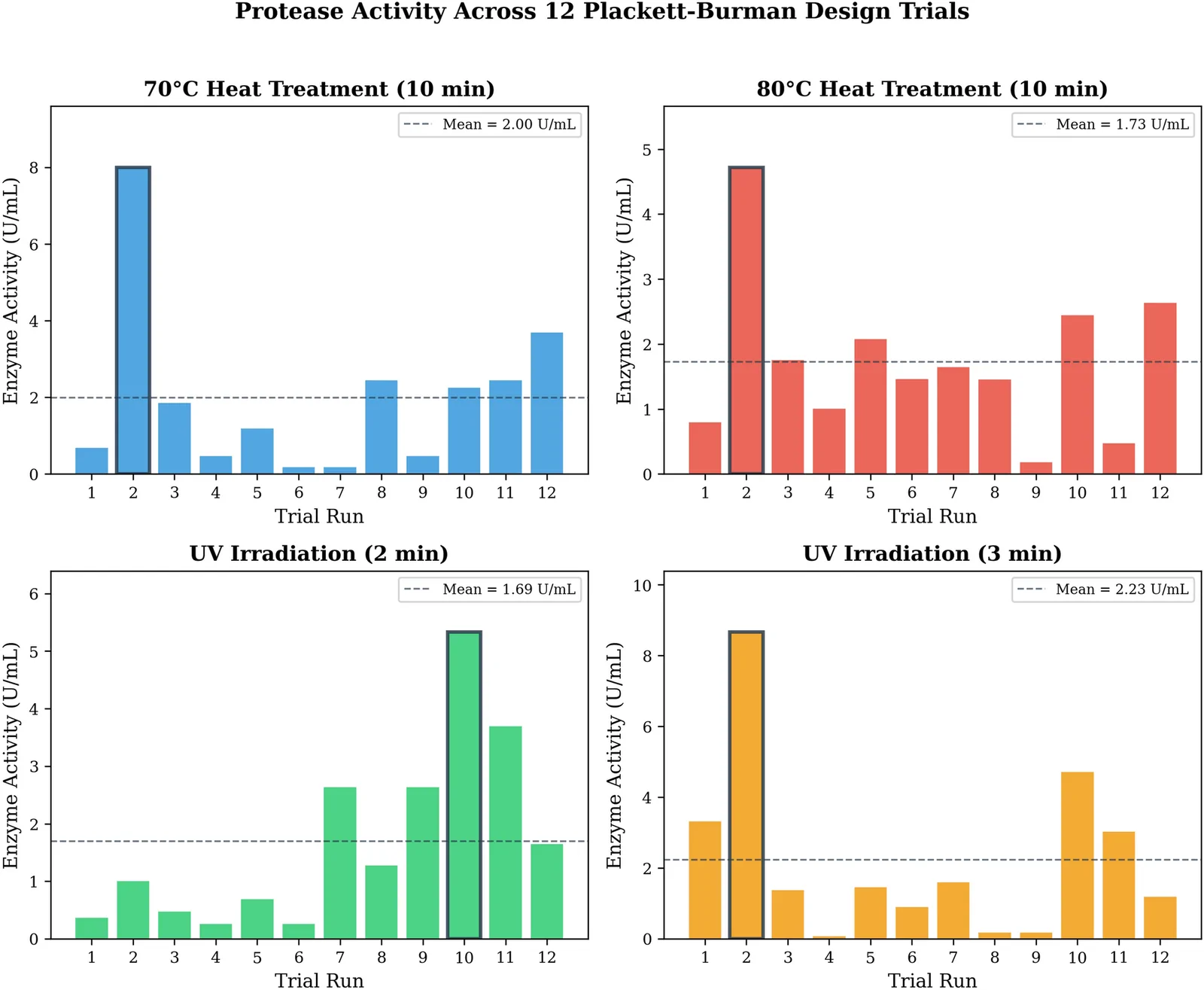
Protease activity (U/mL) across the 12 PBD trial runs under four mutagenic treatments: **a** 70 °C for 10 min, **b** 80 °C for 10 min, **c** UV-2 min, **d** UV-3 min. Horizontal dashed lines show the mean activity for each treatment. Bold-outlined bars mark the run with the highest activity in each panel

**Fig. 3:**
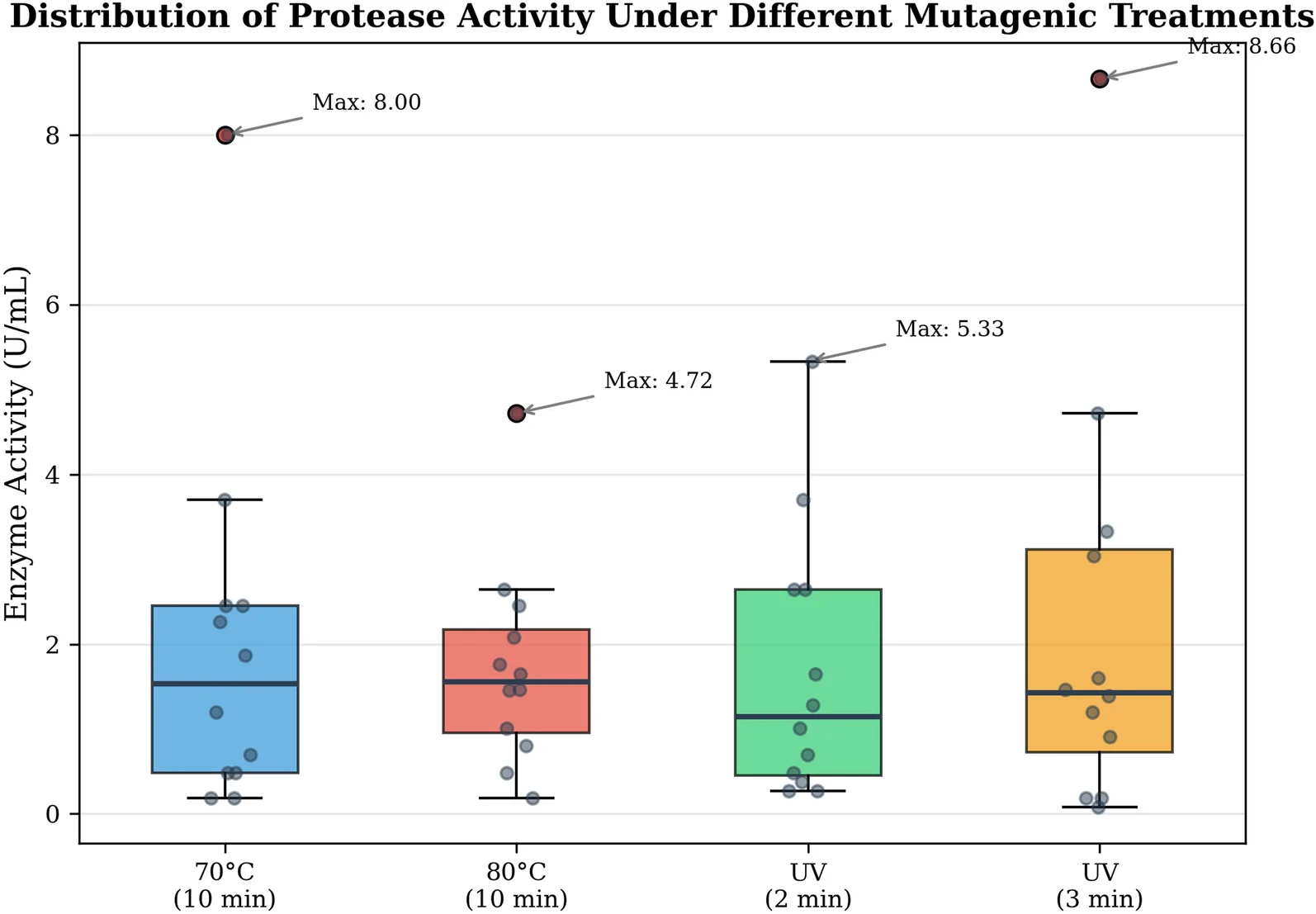
Box-and-whisker plot comparing protease activity distributions across the four mutagenic treatments. Individual data points (n = 12 per treatment) are overlaid on each box. Whiskers extend to 1.5× the interquartile range. Maximum values are annotated

Across the 12 PBD runs, enzyme activity varied widely under each mutagenic treatment, pointing to a strong dependence on both medium composition and stress type (Figs. 2 and 3). Under the 70 °C treatment, Run 2 gave the peak activity of 8.0 U/mL (starch, yeast extract, K_2_HPO_4_ 0.05 M, KH_2_PO_4_ 0.05 M, (NH_4_)_2_SO_4_ 0.05 M, NaNO_3_ 0.1 M, MgSO_4_·7H_2_O 0.002 M, CaCl_2_ 0.002 M, pH 9, 120 rpm); the mean across all runs was 1.996 ± 2.100 U/mL. At 80 °C the peak dropped to 4.72 U/mL (again Run 2), with a tighter spread (mean 1.725 ± 1.151 U/mL), suggesting that the harsher thermal stress narrowed the enzyme response window.

UV-2 min treatment shifted the peak to Run 10 (5.33 U/mL), while Run 2 yielded only 1.01 U/mL—a pattern quite unlike either thermal treatment. The 3-min UV exposure produced the overall highest activity of 8.66 U/mL in Run 2 (mean 2.230 ± 2.361 U/mL). Relative to the unsupplemented basal medium (0.42 ± 0.08 U/mL), the best UV-3 min formulation represented a 20.6-fold improvement, and the best 70 °C formulation a 19.0-fold increase. In terms of volumetric productivity, the UV-3 min optimum reached 0.361 U/mL/h and the 70 °C optimum 0.333 U/mL/h over the 24 h fermentation period. Starch consistently outperformed glucose, in agreement with earlier work showing that slowly metabolizable polysaccharides can alleviate catabolite repression of protease genes in *Bacillus* (Boominadhan et al. 2009). Ammonium sulphate supplementation did not suppress protease output, a finding consistent with reports that ammonium salts lack the repressive effect seen with some other nitrogen sources (Kumar and Takagi 1999).

### ANOVA, main effects, and pareto ranking

**Fig. 4:**
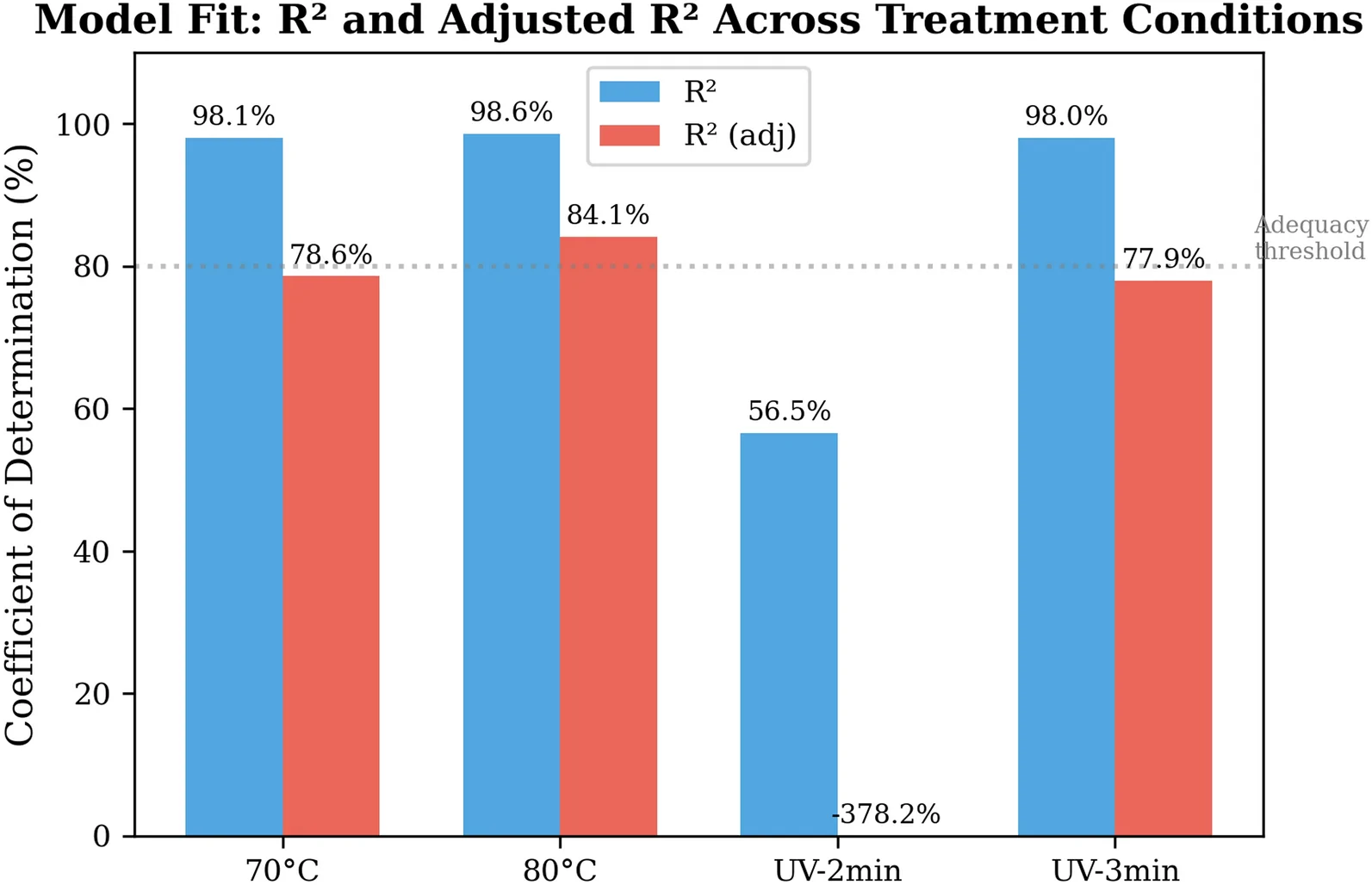
R2 (grey bars) and adjusted R2 (red bars) for the PBD models under each treatment. The horizontal dashed line at 80% marks a conventional adequacy threshold. *For the UV-2 min treatment, adjusted R2 was computed as a negative value, indicating model inadequacy; it is reported here as 0.00% following standard convention

First-order PBD models fitted well for three of the four treatments: 70 °C (R2 = 98.05%, R2adj = 78.58%, F = 5.04), 80 °C (R2 = 98.55%, R2adj = 84.09%, F = 6.81), and UV-3 min (R2 = 97.99%, R2adj = 77.94%, F = 4.89). The UV-2 min model was statistically inadequate (R2 = 56.52%, R2adj = 0.00%, F = 0.13) and biological interpretation of this treatment was therefore not pursued (Fig. 4). The relatively modest adjusted R2 values (77–84%) despite high R2 reflect the limited residual degrees of freedom (df = 1) inherent in a 12-run PBD screening 10 variables. In such saturated designs, two-factor interactions are aliased with main effects, meaning that some of the estimated main effects may partly absorb confounded interaction contributions (Saxena and Singh 2010). This is an intrinsic limitation of screening designs and emphasizes the need for follow-up response surface studies with adequate degrees of freedom.

**Fig. 5:**
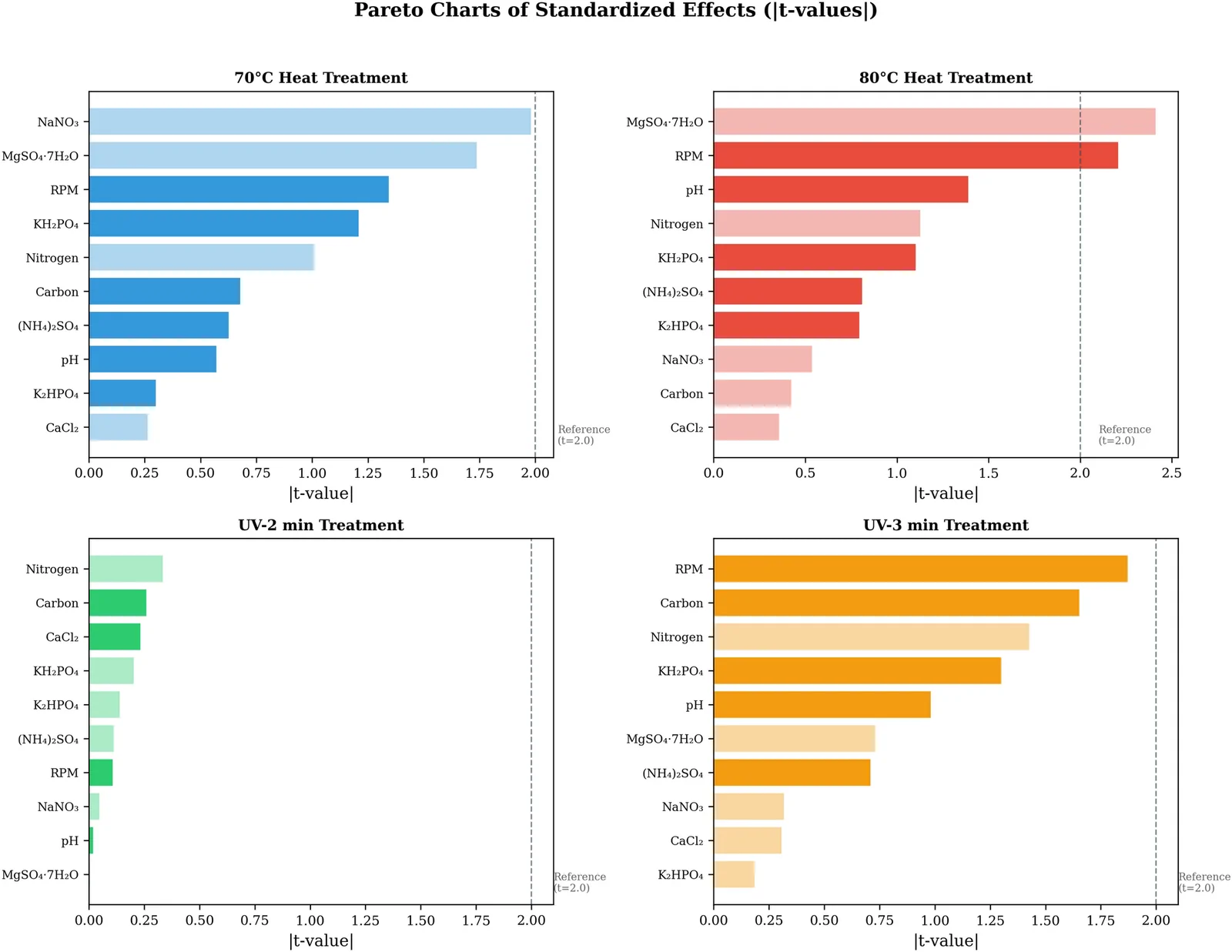
Pareto charts showing the absolute t-values for each factor under the four treatments. Darker bars indicate positive main effects (high level preferred); lighter bars indicate negative effects (low level preferred). The vertical reference line at |t| = 2.0 is shown for visual comparison only and does not represent a formal significance threshold given the single residual degree of freedom

**Fig. 6:**
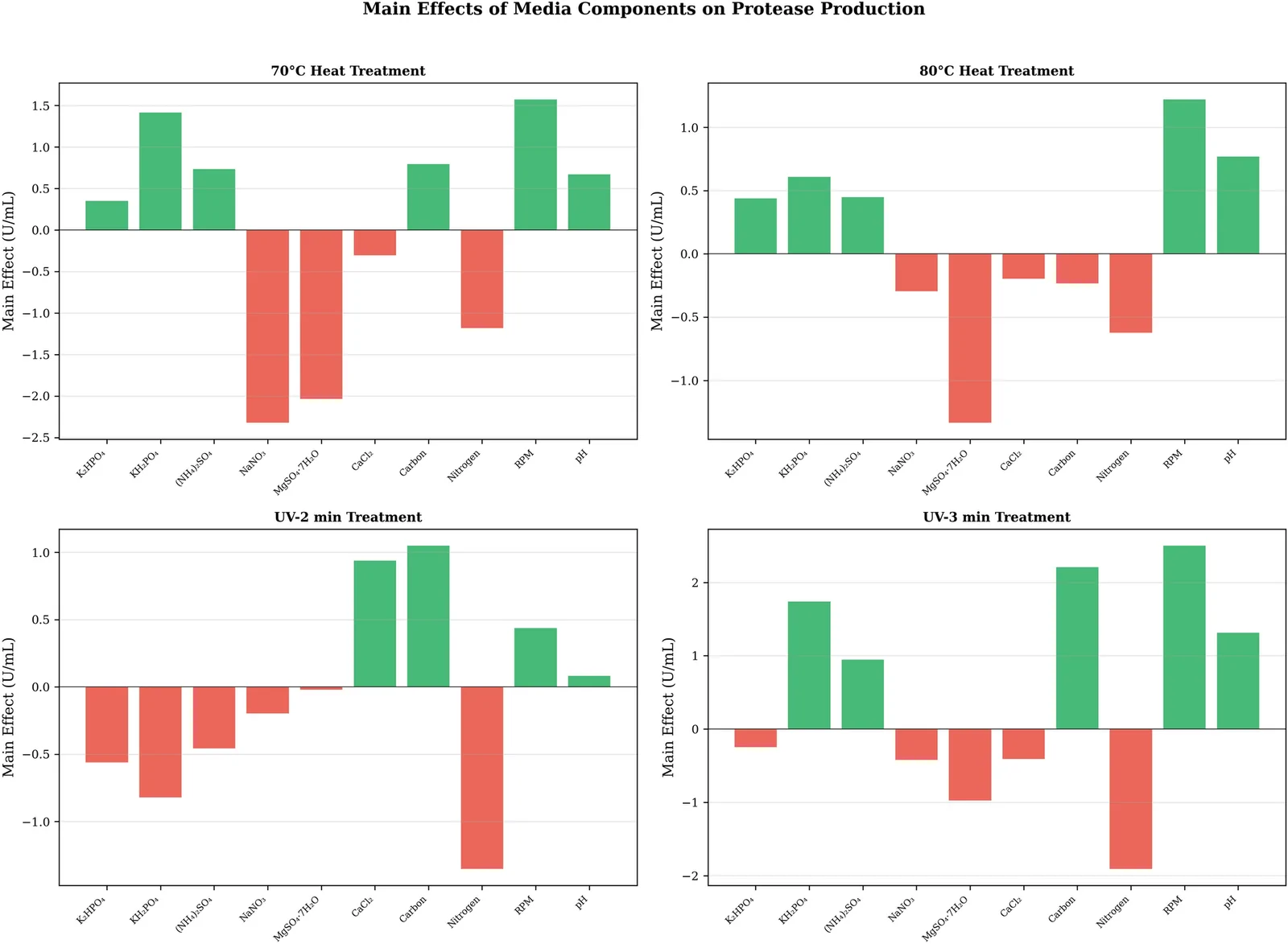
Main effects of the ten medium and process variables on protease activity. Green bars denote a positive effect (high level preferred); red bars a negative effect (low level preferred). Each panel corresponds to one mutagenic treatment

**Fig. 7:**
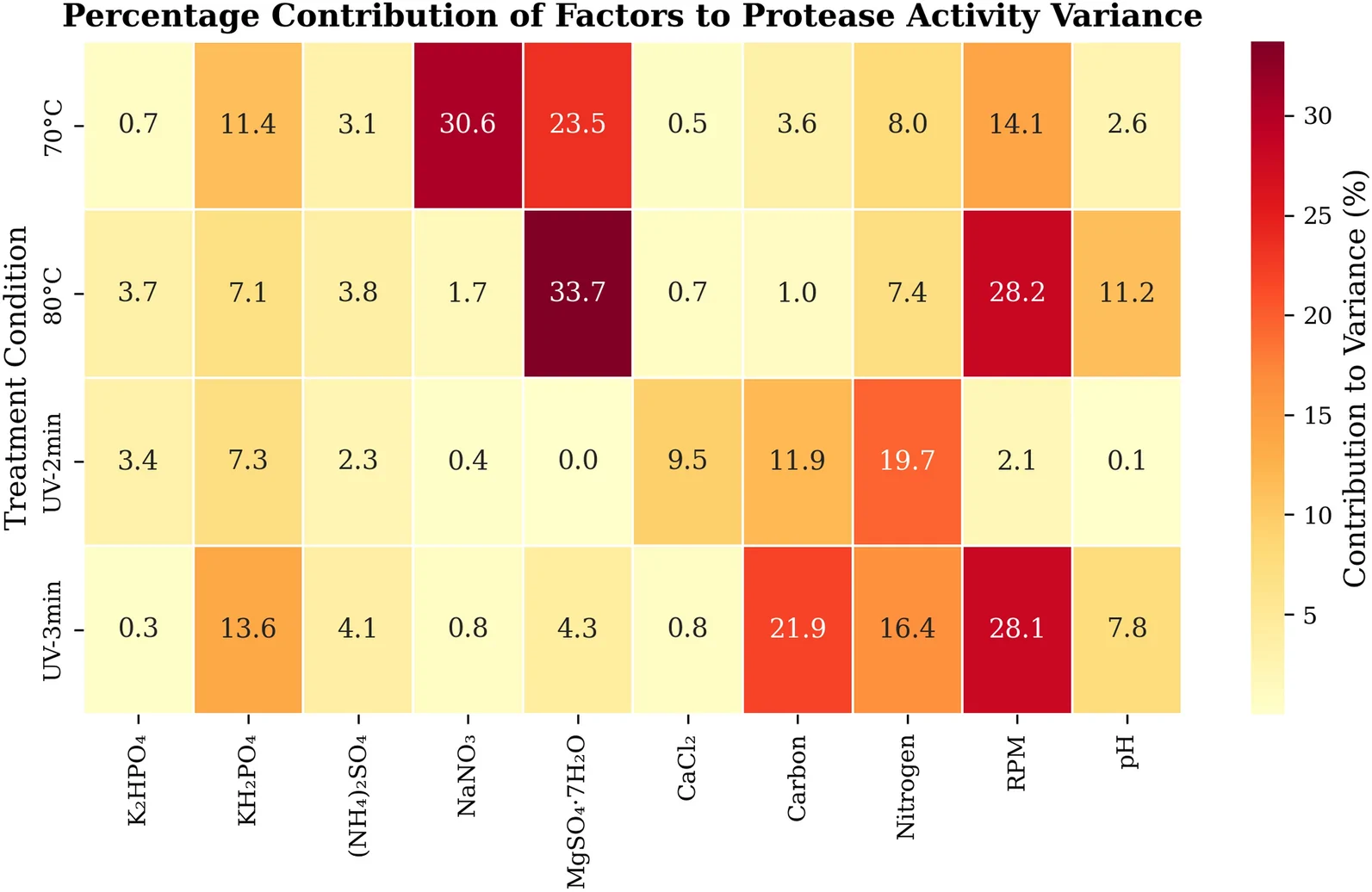
Heatmap of the percentage contribution of each factor to the total sum of squares for protease activity under the four mutagenic treatments. Higher values (darker cells) indicate greater influence on the response

Pareto ranking (Fig. 5) and main effects plots (Fig. 6) identified NaNO_3_, MgSO_4_·7H_2_O, agitation speed, and carbon source as the leading factors across the three adequate models. NaNO_3_ and MgSO_4_·7H_2_O together accounted for over 54% of the variance at 70 °C, while agitation speed and carbon source dominated the UV-3 min response (> 50% combined). The percentage contributions of all ten factors are visualized in Fig. 7. Metal ion supplementation at the lower tested concentrations (MgSO_4_·7H_2_O 0.002 M; CaCl_2_ 0.002 M) was associated with higher protease activity, broadly consistent with earlier reports that moderate divalent cation levels can favour protease stability without impairing secretion (Kaur et al. 2001; Uyar et al. 2011).

### Physiological interpretation of factor effects

The negative effect of NaNO_3_ across the thermal treatments (t = − 1.98 at 70 °C) is consistent with the well-documented nitrogen catabolite repression (NCR) mechanism in Bacillus. In this model, the global transcriptional repressor CodY, responding to intracellular GTP and branched-chain amino acid levels, binds the aprE and nprE promoters and dampens their transcription when nitrogen availability is high (Kumar and Takagi 1999; Strauch and Hoch 1993). Our observation that lower NaNO_3_ concentrations (0.1 M) favoured higher protease titres aligns with this regulatory picture, although direct confirmation would require gene expression studies.

MgSO_4_·7H_2_O had a negative effect in all treatments, most pronounced at 80 °C (t = −2.41). While Mg^2+^ is a cofactor for many cellular enzymes and plays a role in ribosome stability, elevated concentrations have been reported to interfere with extracellular protein translocation and membrane organization in certain Bacillus strains (Akel et al. 2009; Nadeem et al. 2007). The 0.002 M concentration identified here as preferable may represent a balance between adequate cofactor supply and avoidance of secretory impairment, though this interpretation remains to be tested directly.

Agitation speed was consistently positive (t = 1.35–2.21). Higher agitation (120 rpm) would be expected to improve dissolved oxygen transfer and nutrient mixing, both of which are important for aerobic Bacillus metabolism and ATP-dependent protein export (Navaneeth et al. 2009; Patel et al. 2005). These observations agree with those of Patel et al. (2011) and Romsomsaa et al. (2010) who also found agitation to be a significant factor in PBD-based protease screening.

The preference for starch over glucose as carbon source may be explained by the CcpA-mediated carbon catabolite repression (CCR) pathway described in Bacillus (Deutscher 2008). Glucose is known to activate the CcpA-HPr(Ser-P) complex, which binds cre sites near protease gene promoters. Starch, as a slowly hydrolysed polysaccharide, would not trigger the same level of CcpA-mediated repression (Boominadhan et al. 2009; Deutscher 2008). Alkaline pH (pH 9) had a positive effect under all conditions, consistent with the known pH optimum of Bacillus alkaline serine proteases whose catalytic mechanism is most efficient between pH 8 and 11 (Rao et al. 1998; Joo et al. 2002).

### Inter-treatment correlations and stress–response pathways

**Fig. 8:**
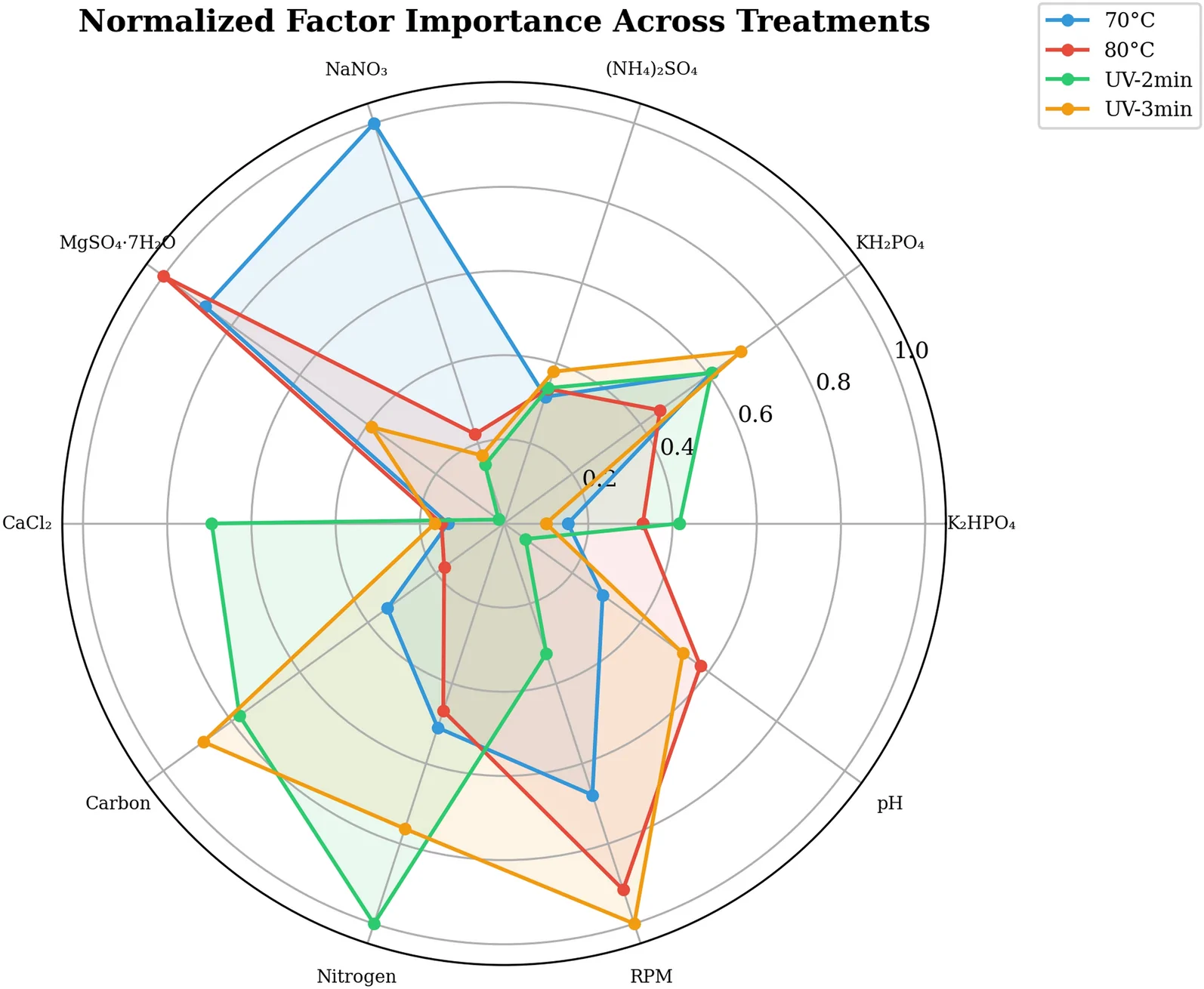
Radar chart of normalized factor importance (|Effect|/max|Effect|) across the four treatments. Each axis represents one of the ten PBD factors; each coloured trace represents one treatment. Larger values indicate greater relative influence

Pearson correlation was strong between 70 and 80 °C responses (r = 0.827), between 70 °C and UV-3 min (r = 0.775), and between 80 °C and UV-3 min (r = 0.718). UV-2 min showed negligible correlation with the thermal treatments (r = 0.046 and − 0.055) (Fig. 8). The high correlations among the three adequate models suggest that these stressors may engage overlapping parts of the Bacillus stress regulon. Both heat shock and prolonged UV exposure have been reported to activate the σ^B^-dependent general stress response, a regulon of over 150 genes covering protein quality control, oxidative stress defence, and extracellular enzyme production (Haldenwang 1995; Völker et al. 1994). Both classes of stress also induce Clp proteases (ClpP, ClpC), whose degradation of misfolded intracellular proteins could increase the amino acid pool available for de novo protease synthesis (Msadek et al. 1998b). These parallels offer a plausible, though so far unverified, explanation for the correlated enzyme responses seen in this study. The negligible UV-2 min correlations are in line with the poor model fit for that condition and likely reflect sub-threshold damage insufficient to trigger a uniform regulatory response across the population (Das and Prasad 2010).

For the consensus optimal formulation (Run 2 conditions), the first-order PBD model predicted a protease activity of 7.84 U/mL under UV-3 min treatment, while the experimentally observed value was 8.66 U/mL, yielding a prediction error of 9.5%. Although this deviation is within acceptable limits for a screening design, it underscores the absence of interaction and quadratic terms that would be captured by a full RSM model. Independent triplicate fermentation runs in the 15 L bioreactor under Run 2 conditions gave a coefficient of variation (CV) of 8.3%, indicating reasonable reproducibility for an unstirred biological system. The maximum protease activities reported here (8.66 U/mL under UV-3 min; 8.0 U/mL at 70 °C) are comparable to values in similar PBD-based Bacillus studies: 7.2 U/mL for B. mojavensis (Beg et al. 2003), 6.8 U/mL for B. subtilis MTCC 441 (Navaneeth et al. 2009), and 8.4 U/mL for B. cereus CA15 (Uyar et al. 2011). A similar PBD-RSM approach was employed by Zhang et al. (2023) for alkaline protease production in recombinant B. licheniformis.The entire screening exercise required only 48 assay points (12 runs × 4 treatments) to evaluate 10 variables—a practical advantage over classical OVAT approaches.

Several limitations should be noted. First, PBD is a screening tool and captures only main effects; factor interactions and curvature in the response surface cannot be estimated. Central composite or Box–Behnken designs are needed to map the full response landscape around the significant factors identified here. Second, we did not perform quantitative survival analysis (colony-forming unit counts) after mutagenic treatments. Consequently, we cannot rule out that observed differences in protease output partly reflect differential survival rather than genuine mutagenic enhancement. Third, the triplicate measurements reported are technical replicates from the same fermentation supernatant, not independent biological replicates; biological replication would strengthen the statistical confidence of the results. Fourth, all mechanistic interpretations—CodY regulation, CcpA repression, σ^B^ activation, Sec pathway involvement, Clp protease activity—are hypotheses grounded in the published literature, not conclusions drawn from molecular experiments performed in this study. Gene expression or proteomic analyses would be required to verify these proposed mechanisms. Finally, the study employed a single fermentation batch per PBD run per treatment, and independent replicate fermentations would further validate the observed trends.

## Conclusions

A 12-run Plackett–Burman Design efficiently screened ten variables for protease production by *Bacillus* sp. MTCC 511 under four mutagenic conditions. ANOVA confirmed R2 > 97% for the 70 °C, 80 °C, and UV-3 min treatments. Agitation speed, NaNO_3_ concentration, MgSO_4_·7H_2_O, and carbon source were the factors with the largest effects, whose behaviour is consistent with established regulatory mechanisms in Bacillus including nitrogen catabolite repression, carbon catabolite repression, and oxygen-dependent secretion. The highest protease activity (8.66 U/mL, a 20.6-fold improvement over the basal medium) was observed under UV-3 min mutagenesis at pH 9 and 120 rpm. Correlation analysis pointed to shared stress–response pathways across thermal and UV-3 treatments. This screening study provides a foundation for subsequent response surface optimization and molecular characterization of the mutagenized strains.

## Data Availability

All data generated during this study are included in the article.
